# Fitness of Pandemic H1N1 and Seasonal influenza A viruses during Co-infection: Evidence of competitive advantage of pandemic H1N1 influenza versus seasonal influenza

**DOI:** 10.1371/currents.RRN1011

**Published:** 2009-08-26

**Authors:** Daniel Roberto Perez, Erin Sorrell, Matthew Angel, Jianqiang Ye, Danielle Hickman, Lindomar Pena, Gloria Ramirez-Nieto, Brian Kimble, Yonas Araya

**Affiliations:** ^*^University of Maryland, College Park; ^†^University of Maryland; ^‡^Virginia-Maryland Regional College of Veterinary Medicine; ^¶^Virginia-Maryland Regional College of Veterinary Medicine-Maryland Campus and ^#^Universidad Nacional De Colombia

## Abstract

On June 11, 2009 the World Health Organization (WHO) declared a new H1N1 influenza pandemic. This pandemic strain is as transmissible as seasonal H1N1 and H3N2 influenza A viruses. Major concerns facing this pandemic are whether the new virus will replace, co-circulate and/or reassort with seasonal H1N1 and/or H3N2 human strains. Using the ferret model, we investigated which of these three possibilities were most likely favored. Our studies showed that the current pandemic virus is more transmissible than, and has a biological advantage over, prototypical seasonal H1 or H3 strains.

## Introduction

    The swine-origin H1N1 pandemic virus was initially linked to human outbreaks in North America in April 2009 and has since spread to over 140 countries in the last five months (http://www.who.int/csr/disease/influenza/en/).  The WHO’s decision to proceed to Phase 6, pandemic influenza, was mainly due to the transmissibility, not the morbidity, of the virus.  This virus has continued to be isolated during the summer months in North America and spread to the regions of the southern hemisphere where the influenza season is at its peak.  Numbers from South American countries indicate this pandemic strain predominates over the seasonal H1 and H3 strains, although all 3 strains appear to circulate. Studies to determine the possibility of reassortment following co-infection of an avian and human virus has been reported [1], as have naturally occurring co-infections in the human population [2] [3] [4] . However, to date there has been no report of co-infection between swine-origin and seasonal human influenza A viruses.

## Results and Discussion

### Effects of co-infection of pandemic influenza and seasonal strains in the ferret model

    To establish whether the current pandemic H1N1 virus has a biological advantage over the seasonal H1N1 and/or H3N2 strain(s) and whether reassortment between the pandemic and seasonal strains is rapidly favored *in vivo*, we performed co-infection studies in ferrets.  The ferret is a well-established model for human influenza infection, pathogenesis and transmission studies [5] [6] .  Ferrets infected with either seasonal H1N1 (A/Brisbane/59/07) [BR/59] or seasonal H3N2 (A/Brisbane/10/07) [BR/10] virus supported replication and transmission to both direct and respiratory droplet contacts (Fig 1B, D and data not shown).  All ferrets shed virus for 5-7 days and seroconverted (Table 1 and data not shown). However, A/California/04/09 (H1N1) [Ca/04], an early representative isolate of the pandemic strain, replicated to higher titers, caused more severe clinical signs, and transmitted to direct and respiratory droplet contact ferrets more efficiently when compared to either seasonal strain (Fig 1A).  Tissue tropism studies indicated that both seasonal strains and the pandemic strain were isolated at comparable titers in the nasal turbinate however virus isolated from the trachea of either seasonal strain was on average two folds lower than the pandemic H1N1.  Only one ferret infected with BR/59 had detectable virus in the lungs, albeit to titers 3 logs lower than the pandemic virus (Fig 2A).  In summary, Ca/04 was isolated in all respiratory tissues sampled and was present at higher titers than either seasonal H1N1 or H3N2 strain, which were only consistently isolated in the nasal turbinate and trachea (Fig 2A). The Ca/04 virus also caused more congestion leading to bronchopneumonia and infiltration of inflammatory cells compared to the seasonal strains (Fig 2B-I).

**Figure:**
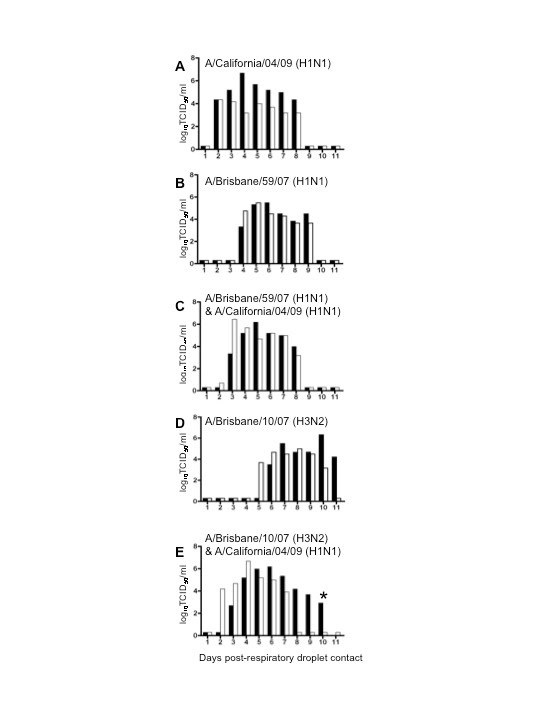

#### Figure 1.  Influenza respiratory droplet transmission in single and co-infection studies. 

*Ferrets were inoculated with 10^6^ TCID_50_ of the corresponding virus combination. Direct contact ferrets and respiratory droplet contact ferrets were introduced at 24 h.p.i. Black and white bars represent individual ferrets. Respiratory droplet contacts shown: (A) A/California/04/09 [Ca/04] H1N1 virus, (B) A/Brisbane/59/07 [BR/59] H1N1, (C) co-infected with BR/59 and Ca/04, (D) A/Brisbane/10/07 [BR/10] H3N2, (E) or co-infected with BR/10 and Ca/04 (E). In (E), day 10 p.c., the respiratory contact from the group represented by the black bars died, as noted by a star in the bar graph. Post-mortem examination indicated intestinal hemorrhage and virus was isolated from respiratory tissues. Titers are expressed as log_10_ values of TCID_50_/ml with the limit of detection at 0.699 log_10_TCID_50_/ml. Viruses were grown and titrated in MDCK cells as previously described [6][7]. All experiments with live virus were performed in an animal biosafety level-3+ containment facility.*

**Figure:**
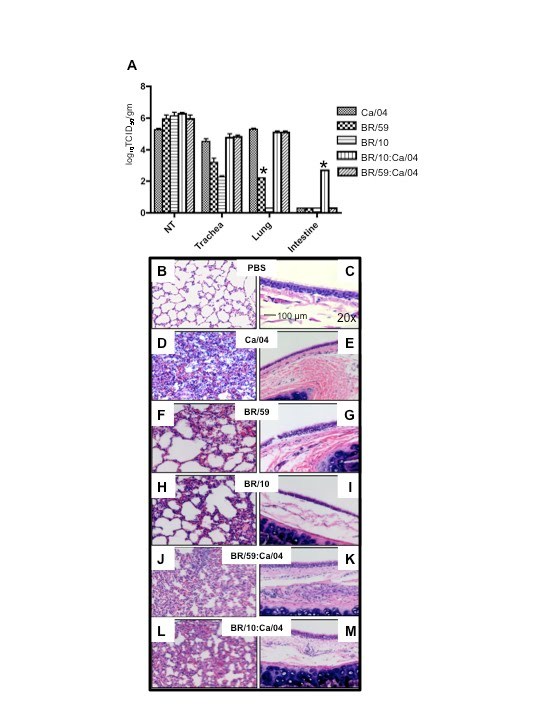

#### Figure 2. Growth and pathology of pandemic and seasonal influenza viruses in the ferret. 

(A) Ferrets were infected with 10^6^ TCID50 of either Ca/04, BR/59, BR/10 or nasal washes from the last day of respiratory droplet contact shedding in the co-infected groups (passed once in MDCKs to achieve a titer of 10^6^ TCID50). Tissues were collected 4 dpi and samples were taken for histopathology and to determine titers and tropism. Nasal turbinate, trachea, lung, intestine, liver, spleen, heart and olfactory bulb were homogenized and titrated in MDCKs. 10% (w/v) of the homogenates were used to determine viral titers in MDCK cells. Abbreviations on figure include: NT, nasal turbinate. Asterisk indicates when only one out of two ferrets was positive for virus. For histopathology studies tissues were paraffin-embedded and stained for hematoxylin and eosin. PBS (B,C), Ca/04 (D,E), BR/59 (F,G), BR/10 (H,I), BR/59:Ca/04 [H1/H1] (J,K), BR/10:Ca/04 [H3/H1] (L,M). Lung (B,D,F,H,J,L) and trachea (C,E,G,I,K,M) samples are shown.

**

#### Table 1. HI titers pre- and post-infection of single and co-infection transmission studies

**Table:** 

**BR/59 (H1N1)**	**Infected**		**Direct Contacts**		**Respiratory Droplet Contacts**	
**VIRUS**	0 dpi	15 dpi	0 dpi	15 dpi	0 dpi	15 dpi
**BR/59 H1N1**	20, 40	1280, 1280	20, 20	1280, 1280	20, 40	1280, 1280
**BR/10 H3N2**	40, 40	40, 40	20, 40	20, 40	40, 40	40, 40
**Ca/04 H1N1**	< 10, 10	< 10, 10	< 10, 10	< 10, 10	< 10, 10	< 10, 10
** **						
**BR/10 (H3N2)**	**Infected**		**Direct Contacts**		**Respiratory Droplet Contacts**	
**VIRUS**	0 dpi	15 dpi	0 dpi	15 dpi	0 dpi	15 dpi
**BR/59H1N1**	20, 20	20, 20	20, 40	20, 40	20, 10	20, 10
**BR/10H3N2**	20, 40	2560, 1280	40, 40	1280, 2560	40, 80	2560, 2560
**Ca/04H1N1**	< 10, 10	< 10, 10	< 10, 10	< 10, 10	< 10, 10	< 10, 10

****

**Table:** 

**BR/59:Ca/04 (H1:H1)**	**Infected**		**Direct Contacts**		**Respiratory Droplet Contacts**	
**VIRUS**	0 dpi	15 dpi	0 dpi	15 dpi	0 dpi	15 dpi
**BR/59 H1N1**	40,40	1280,640	20,40	20,80	40,40	40,40
**Ca/04 H1N1**	<10,10	2560,5120	<10,10	640,2560	<10,10	2560,2560
**BR/10 H3N2**	80,40	80, 40	20,40	20,40	40,80	40,80
** **						
**BR/10:Ca/04 (H3:H1)**	**Infected**		**Direct Contacts**		**Respiratory Droplet Contacts**	
**VIRUS**	0 dpi	15 dpi	0 dpi	15 dpi	0 dpi	15 dpi
**BR/59 H1N1**	40,20	40,20	20,20	20,20	20,20	20,20
**Ca/04 H1N1**	<10,10	640,5120	<10,10	1280,2560	<10,10	2560,2560
**BR/10 H3N2**	80,80	320,640	80,80	160,160	80,80	320,80

    To determine the possibility of co-infection of pandemic and seasonal influenza viruses, ferrets were either co-infected with Ca/04 H1N1 and the seasonal BR/59 H1N1 virus [BR/59:Ca/04] or co-infected with Ca/04 and the seasonal BR/10 H3N2 virus [BR/10:Ca/04].  Successful co-infection was confirmed through PCRs performed directly on nasal washes collected 1-day post-infection (p.i.) with virus-specific primers (Fig. 3C,F), as well as through seroconversion against both viruses in inoculated ferrets (Table 1).  Transmission to direct and respiratory droplet contact ferrets was observed in both co-infections (Fig 1C, E and data not shown).  In each case, co-infection led to an intermediate transmission phenotype where virus transmitted to direct and respiratory droplet contacts earlier than the seasonal strain and within a day of the pandemic strain.  In addition, ferrets shed viral titers higher than the seasonal strain but comparable to the pandemic strain.  Clinical signs including onset of sneezing and body temperatures were similar in the co-infected groups; however, ferrets from the BR/59:Ca/04 [H3/H1] group presented with diarrhea and weight loss and one respiratory droplet contact had to be euthanized while still shedding virus (Fig 1E).  Tissues collected from the euthanized ferret indicated that virus was present in the nasal turbinate, trachea, lung and olfactory bulb (data not shown). 

**Figure:**
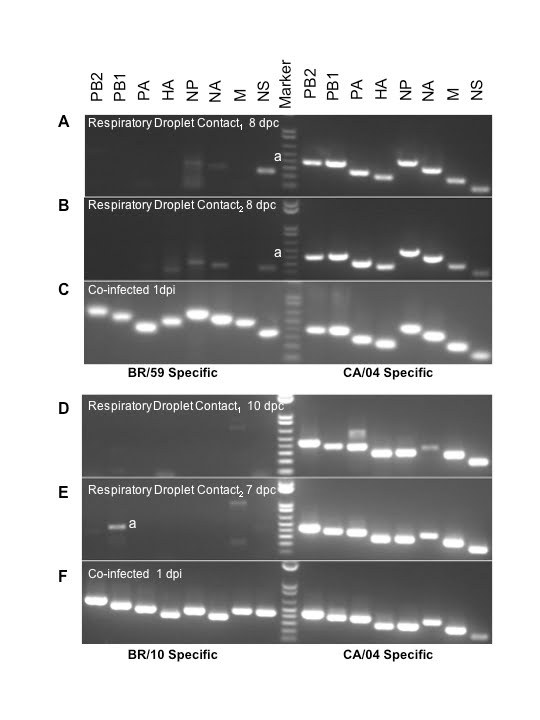

#### Figure 3. RTPCR for co-infected BR/59:Ca/04 (H1:H1) and BR10:Ca/04 (H3:H1) respiratory droplet contacts.  

*Nasal washes from co-infected ferrets (infected, direct contact, respiratory droplet contact) were collected days 1-11 p.i.  Nasal washes from respiratory droplet contacts first and last day of shedding were analyzed by PCR using primers specific for BR/59, BR/10, and Ca/04.  Data for the last day of shedding from each respiratory droplet contact is shown for the BR/59:Ca/04 (H1:H1) pair day 8 post-contact (A, B) and the BR10:Ca/04 (H3:H1) pair days 7 and 10 post-contact (D, E).  Nasal wash from ferrets directly inoculated with BR/59:Ca/04 (H1:H1) and BR10:Ca/04 (H3:H1) virus combinations were analyzed 1 dpi to confirm efficient replication and shedding of both viruses (C, F, respectively).* *^a^* *denotes non-specific binding confirmed by sequencing the PCR product to eliminate suspicion of reassortment.*

### No evidence of reassortment between pandemic influenza H1N1 strain and seasonal influenza strains

    To establish the virus population(s) selected for respiratory droplet transmission following co-infection in both BR/59:Ca/04 [H1/H1] and BR/10:Ca/04 [H3/H1] groups, viral RNA was purified and RTPCRs were performed directly from the nasal washes collected from the respiratory droplet contacts.  We selected the first and last days of viral shedding as the most fit virus population to a) transmit to respiratory droplets, b) to survive transmission, and c) to be passed on in respiratory droplet form.  We performed RTPCRs on purified viral RNA from nasal washes collected from respiratory droplet contacts and/or stock virus used in our single Ca/04, BR/59, and BR/10 infection studies, as controls for virus-specific primers. In addition, virus in nasal washes from co-infected and single infected ferrets in the respiratory droplet contact groups were amplified once in cell culture (P1 MDCK) to determine which type of virus population was prevalent. Viruses isolated from the first and last days of shedding from respiratory droplet contacts co-infected with either BR/59:Ca/04 (H1/H1) or BR/10:Ca/04 (H3/H1) show a single virus population, a full Ca/04 virus. The data was consistent regardless of whether we analyzed RNA obtained directly from nasal washes or P1 MDCK passage virus (last day shown, Fig 3A, B, D, E and data not shown.)  In contrast, directly inoculated ferrets with either the BR/59:Ca/04 (H1/H1) or BR/10:Ca/04 (H3/H1) virus combinations showed both virus populations at 1 dpi, from either nasal washes or P1 MDCK grown virus (Fig 3C and F).  HI analysis of the BR/59:Ca/04 (H1/H1) ferret sera indicated that only inoculated ferrets had an antibody response to the seasonal BR/59 while all ferrets had strong responses to Ca/04, suggesting that the Ca/04 out-competes the seasonal H1N1 virus, most likely at direct transmission (Table 1). Interestingly, although a majority of the ferret sera from the BR/10:Ca/04 (H3/H1) group responded to both BR/10 and Ca/04 (Table 1), only Ca/04 was isolated in all nasal washes and organs tested (Fig 3D and E, organ data not shown). To further determine the nature of the predominant virus population in respiratory droplet contacts in the co-infected groups, the nasal washes from the respiratory droplet contact ferrets, collected on the last day of shedding (P1 MDCKs), were used to infect a new set of ferrets and determine which virus population(s) prevailed (Fig 2A). Nasal washes and respiratory tissues collected from this new set of ferrets revealed the presence of high virus titers comparable to Ca/04 single infection (Fig 2A, nasal wash not shown). Interestingly, PCR analysis of tissue homogenates (nasal turbinate, trachea, lung, and intestine) from this set of ferrets revealed the presence of Ca/04 and no evidence of BR/59 or BR/10 (not shown).  In addition, histopathology of lungs and trachea from both co-infected groups show similar bronchopneumonia and inflammatory infiltration like Ca/04 single infection (Fig. 2J-M).

    These studies emphasize the notion that the pandemic strain has growth and transmissibility advantages over the seasonal strains.  More importantly, no evidence of reassortment was detected in PCRs performed on direct nasal washes or nasal washes passed once in MDCK cells in either of the respiratory droplet contacts from either co-infection group (Fig. 3). It should be noted that the pre-infection HI titers for both the H1 and H3 seasonal strains were minimal, although detectable, in all ferrets (Table 1). Thus, we cannot exclude the possibility that the relative transmissible advantage of the Ca/04 H1N1 is merely a consequence of prior immunity in ferrets against the seasonal influenza strains.  Our data, however, clearly showed that both the BR/59 (H1N1) and BR/10 (H3N2) replicated and transmitted well in single virus infections.  It remains to be determined whether the minimal antibody levels in ferrets against the seasonal flu strains contributed to the Ca/04’s ability to out-compete them. However, we must note that our studies reflect the most likely scenario in the human population as it is expected that most people carry antibodies against H1 and H3 seasonal strains. This is particularly true in those within the 15-40 age group that appear to have shown more exacerbated disease with the pandemic strain.  Nevertheless, these observations are consistent with the notion that the emergence of novel pandemic strains in humans may be favored, not only because of lack of immunity against the new virus but also because of the presence of immunity against extant seasonal strains.

## Conclusions    

Recent reports have speculated on the possibility that the new pandemic H1N1 strain may become more virulent and/or more transmissible if it were to reassort with seasonal influenza strains [8] [9] [10].  Alternatively, it has been postulated that the new H1N1 virus may not be completely adapted to the human population and that additional changes could lead to more virulent and perhaps more transmissible strains [8] [9] [10]. Our studies show that the pandemic Ca/04 virus has a clear biological advantage in replication, transmission, tropism and pathogenesis when compared to both seasonal H1 and H3 representative strains.  In addition, co-infection of seasonal and pandemic strains did not result in the rapid selection of reassortant viruses that either improved replication or transmission or exacerbated virulence.  Although we must be cautious interpreting studies in the ferret model, it is reasonable to speculate that this prototypical pandemic strain, Ca/04, has all the makings of a virus fully adapted to humans. At present, reassortment may not be particularly favored.  We must note that more analysis need to be done to rule the possibility that either the inoculated, direct contacts and/or respiratory droplet contacts shed a population of reassortant viruses at any time during the infection. Nevertheless, if any reassortant was present, our data clearly indicates that they did not have an obvious biological advantage over the Ca/04 wild type virus during transmission via respiratory droplets. Alternatively, studies looking at additional combinations of pandemic and seasonal strains may provide a better understanding of the reassortment potential among these viruses. We observed that in the BR/10:Ca/04 co-infection group, respiratory droplet contact ferrets seroconverted to both the Ca/04 H1N1 and the BR/10 H3N2 yet, only high levels of Ca/04 replication were detected.  However, since co-infection with the H3 seasonal strain resulted in exacerbation of clinical signs, it is important to determine whether co-infections have been associated in some of the lethal human cases.  Surveillance should consider careful examination of clinical cases and determine whether co-infections are related to the morbidity and mortality associated with the pandemic strain. Based on studies in the ferret model, strong emphasis should be placed on determining whether underlying H3 infections are associated with co-infections with the pandemic strain.

## Materials and Methods

### Viruses and cells. 

    Viruses were grown in Madin-Darby Canine kidney (MDCK) cells and titrated with tissue culture infectious dose 50 (TCID_50_) in MDCKs.  For A/Brisbane/59/07 (H1N1) and A/Brisbane/10/07 (H3N2) the viruses were received as egg passage 1 (EP1) was grown and titrated in MDCKs (EP1/MDCKP1).  For A/California/04/09 (H1N1) virus was received as MDCK P1 and then grown twice in MDCKs for MDCK P3 and titrated.  All experiments with live virus were performed in a biosafety level-3^+^ containment facility. 

### Animal studies. 

    Female Fitch ferrets (4-7 months old), Triple F Farms, Sayre, PA were used for studies.  Animal studies were conducted under guidelines approved by the Animal Care and Use Committees of the University of Maryland and the Centers for Disease Control and Prevention. All animal studies were conducted in a biosafety level-3^+^ containment facility approved by the U.S. Department of Agriculture. Methods of infection, transmission scheme, nasal wash collection and observation of clinical signs was followed as previously described [6] [7] .  Briefly, ferrets were housed in ABSL2 facilities and monitored 5 to 7 days to establish baseline body temperature and overall health.  A subcutaneous implantable temperature transponder (Bio Medic Data Systems, Seaford, DE) was placed in each ferret for identification and temperature readings.  Temperatures were recorded daily and three days before infection blood was collected and serum tested for antibodies against the inoculum virus using the hemagglutinin inhibition (HI) assay.   Titers at or lower than 20-40 were considered “influenza A-free”.

    The basic transmission study scheme consisted of groups of three ferrets: one infected, one direct contact and one respiratory droplet contact, as described previously [7].  Ferrets were lightly anesthetized with ketamine (20mg/kg) and xylazine (1mg/kg) via an intramuscular injection and inoculated intranasally (i.n.) with 10^6^ TCID_50_ of virus in PBS, 250 µl per nostril.  Twenty-four hours later, two naïve ferrets were introduced into the isolator.  A direct contact was introduced into the same cage as the infected ferret while the respiratory droplet contact was placed into a cage separated from the infected and direct contact by a wire mesh wall.  The wire mesh prevented physical contact between the respiratory droplet contact and the infected/direct contact ferrets therefore, only air was shared among the group.  Individual weight and body temperatures were measured daily; fevers were defined as 3 standard deviations above an individual’s baseline temperature.  To monitor viral shedding nasal washes were collected daily for up to two weeks.  Ferrets were anesthetized as described above and 1 ml of PBS was used to induce sneezing.  Nasal washes were immediately tested for virus using the FLU DETECT^TM^ Antigen Capture Test (Synbiotics Corp., San Diego, CA) and additional aliquots were stored at -80^0^C before performing TCID_50_ titrations in MDCK cells.  Two weeks after shedding blood was collected and seroconversion determined by HI Assay. 

### HI assay. 

    Serum samples were collected 14-15 days p.i. and treated with receptor-destroying enzyme (Accurate Chemical and Scientific Corp., Westbury, NY) to remove nonspecific receptors.  The anti-viral antibody titers were evaluated using the HI assay system outlined by the WHO Animal Influenza Training Manual (WHO/CDS/CSR/NCS/2002.5).  HI assays were performed using homologous and heterologous viruses.

### Tissue collection and titration. 

    Groups of two ferrets were inoculated with 10^6^ TCID_50_ of either: A/California/04/09 (H1N1), A/Brisbane/59/07 (H1N1), A/Brisbane/10/07 (H3N2), nasal wash collected from BR/59:Ca/04 co-infected respiratory droplet contacts on day 8 post-contact (p.c) (passed once in MDCK), nasal wash collected from BR/10:Ca/04 co-infected respiratory droplet contacts on days 7 and 10 p.c. (passed once in MDCK), or mock-infected with PBS.  Ferrets were euthanized on day 4 p.i.  Olfactory bulb, nasal turbinate, trachea, lung, heart, spleen, liver and intestine were collected and stored at ─80 ºC for virus titration as previously described [7] . To determine the tissue distribution of the virus, 10% (w/v) of tissue homogenate was prepared with PBS and the viral titers were determined in MDCK cells. For histopathology, paraffin-embedded sections of 5-µm thickness were cut and stained with H&E (Histoserv, Inc., Germantown, MD). Representative microscopic photos were taken with the SPOT ADVANCED software (Version 4.0.8, Diagnostic Instruments, Inc., Sterling Heights, MI).

### Determination of Virus Populations/Genotyping.  

    To establish the virus population(s) during the co-infection transmission studies RNA was collected directly from nasal washes, nasal washes passed once in MDCKs and tissue homogenates (Qiagen, Valencia, CA).  Reverse transcriptase polymerase chain reaction was used to determine the origin of each gene in all samples.  Nasal washes analyzed included: day 1 post-infection (p.i.) from both infected ferrets in both co-infection groups (BR/59:Ca/04 [H1/H1] and BR/10:Ca/04 [H3/H1]), the last day of shedding for each respiratory droplet contact from both co-infected groups (8 days post-contact (pc) from both respiratory droplet contacts in BR/59:Ca/04 [H1/H1] and days 10 and 7 pc from respiratory droplet contacts in BR/10:Ca/04 [H3/H1]).  The aforementioned nasal wash samples from all 4 respiratory droplet contacts (both H1/H1 and H3/H1 groups) were also passed once in MDCK cells to determine viral population after passage in cell culture.  Tissue homogenates collected 4 dpi, described above, were also processed for RNA.

    Primers were designed to specifically hybridize to Ca/04, BR/59 or BR/10 genes and PCR products were visualized on 1% agarose gels.  Primer sequences and reaction conditions are available upon request.  Any background bands were cloned and sequenced to confirm non-specific binding. 

## Acknowledgments

This research was possible through funding by the CDC-HHS grant (1U01CI000355), NIAID-NIH grant, (R01AI052155), CSREES-USDA grant (2005-05523), and NIAID-NIH contract (HHSN266200700010C).  We are indebted to Ivan Gomez-Osorio for his excellent laboratory techniques and animal handling assistance.  We would like to thank Andrea Ferrero and Theresa Wolter for their laboratory managerial skills and the Centers for Disease Control and Prevention, Atlanta, GA, for supplying the wild type viruses used in this study. The opinions of this manuscript are those of the authors and do not necessarily represent the views of the granting agencies. 

## Competing Interests

The authors have declared that no competing interests exist.
